# 3-Ethyl-*cis*-2,6-di­phenyl­piperidine

**DOI:** 10.1107/S1600536813021417

**Published:** 2013-08-17

**Authors:** V. Maheshwaran, S. Abdul Basheer, A. Akila, S. Ponnuswamy, M. N. Ponnuswamy

**Affiliations:** a Centre of Advanced Study in Crystallography and Biophysics, University of Madras, Guindy Campus, Chennai 600 025, India; b Department of Chemistry, Government Arts College (Autonomous), Coimbatore 641 018, India

## Abstract

In the title compound, C_19_H_23_N, the piperidine ring adopts a chair conformation. The phenyl rings at the 2,6-positions of the piperidine ring occupy equatorial orientations. The crystal structure features C—H⋯π inter­actions.

## Related literature

For the biological activity of piperidine derivatives, see: Nalanishi *et al.* (1974 ▶). For the synthesis, see: Ponnuswamy *et al.* (2002 ▶). For puckering parameters, see: Cremer & Pople (1975 ▶) and for asymmetry parameters, see: Nardelli (1983 ▶).
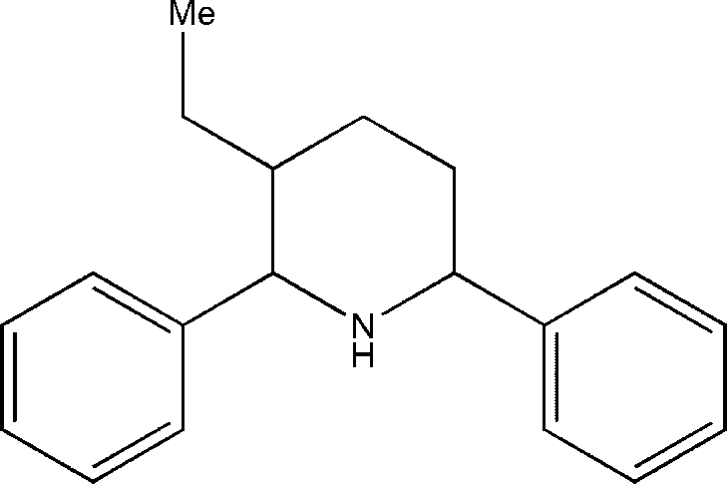


## Experimental

### 

#### Crystal data

C_19_H_23_N
*M* *_r_* = 265.38
Triclinic, 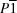

*a* = 5.5384 (5) Å
*b* = 9.2717 (9) Å
*c* = 16.0483 (14) Å
α = 75.508 (5)°
β = 89.474 (5)°
γ = 81.625 (5)°
*V* = 789.04 (13) Å^3^
*Z* = 2
Mo *K*α radiation
μ = 0.06 mm^−1^
*T* = 293 K
0.20 × 0.19 × 0.19 mm

#### Data collection

Bruker SMART APEXII CCD diffractometer
Absorption correction: multi-scan (*SADABS*; Bruker, 2008 ▶) *T*_min_ = 0.987, *T*_max_ = 0.988
11467 measured reflections
3251 independent reflections
2579 reflections with *I* > 2σ(*I*)
*R*_int_ = 0.025

#### Refinement

*R*[*F* ^2^ > 2σ(*F* ^2^)] = 0.043
*wR*(*F* ^2^) = 0.124
*S* = 1.03
3251 reflections
185 parameters
H atoms treated by a mixture of independent and constrained refinement
Δρ_max_ = 0.13 e Å^−3^
Δρ_min_ = −0.18 e Å^−3^

### 

Data collection: *APEX2* (Bruker, 2008 ▶); cell refinement: *SAINT* (Bruker, 2008 ▶); data reduction: *SAINT*; program(s) used to solve structure: *SHELXS97* (Sheldrick, 2008 ▶); program(s) used to refine structure: *SHELXL97* (Sheldrick, 2008 ▶); molecular graphics: *ORTEP-3 for Windows* (Farrugia, 2012 ▶); software used to prepare material for publication: *SHELXL97* and *PLATON* (Spek, 2009 ▶).

## Supplementary Material

Crystal structure: contains datablock(s) global, I. DOI: 10.1107/S1600536813021417/ng5337sup1.cif

Structure factors: contains datablock(s) I. DOI: 10.1107/S1600536813021417/ng5337Isup2.hkl

Supplementary material file. DOI: 10.1107/S1600536813021417/ng5337Isup3.cml

Additional supplementary materials:  crystallographic information; 3D view; checkCIF report

## Figures and Tables

**Table 1:** Hydrogen-bond geometry (Å, °) *Cg*2 and *Cg*3 are the centroids of the C7–C12 and C13–C18 rings, respectively.

*D*—H⋯*A*	*D*—H	H⋯*A*	*D*⋯*A*	*D*—H⋯*A*
C5—H5*B*⋯*Cg*2^i^	0.97	3.14	3.893 (2)	136
C10—H10⋯*Cg*3^ii^	0.93	2.92	3.702 (2)	142
C20—H20*A*⋯*Cg*3^iii^	0.96	3.17	3.926 (2)	137

Symmetry codes: (i) 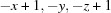; (ii) 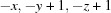; (iii) 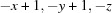.

**Fig. 1.**
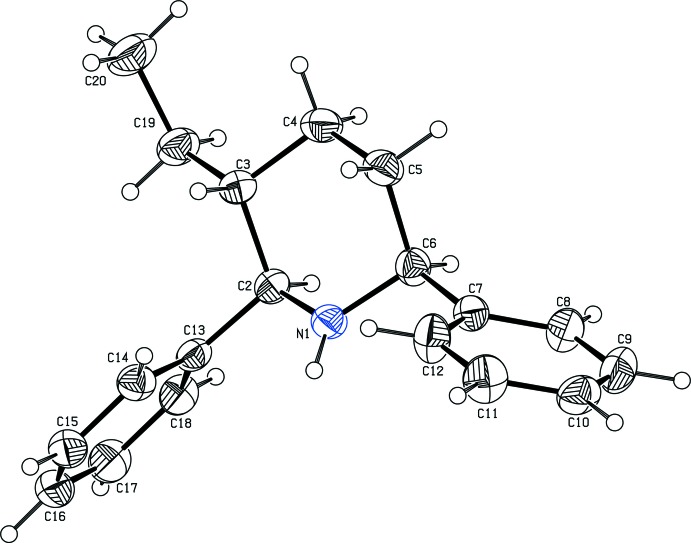
The molecular structure of the title compound, showing the atomic numbering and displacement ellipsoids drawn at 30% probability level.

**Fig. 2.**
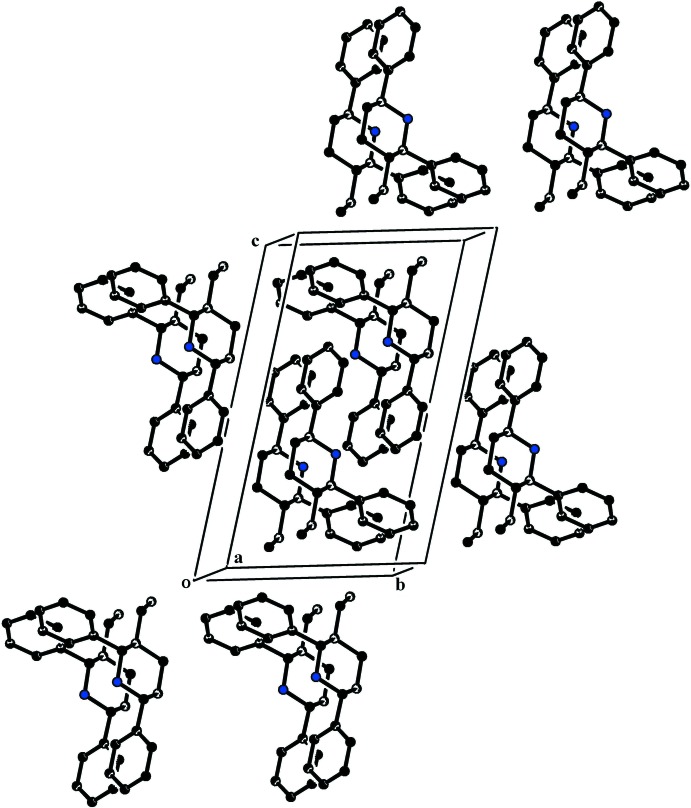
The crystal packing of the molecules viewed down a axis.

## supplementary crystallographic information

### 1. Comment

Piperidine derivatives are the valued heterocyclic compounds in the field of medicinal chemistry. As an example, piperidines have been found to exhibit blood cholesterol-lowering activities (Nalanishi *et al.*, 1974). Against this background and to ascertain the molecular structure and conformation, the X-ray crystal structure determination of the title compound has been carried out.

The *ORTEP* plot of the molecule is shown in Fig. 1. The piperidine ring adopts chair conformation with the puckering parameters (Cremer & Pople, 1975) and the asymmetry parameters (Nardelli,1983) are: q_2_=0.039 (14) Å, q_3_ = 0.576 (14) Å, φ_2_ = 1(2)° and Δ_s_ (N1& C4)= 0.86 (12)°.

The planar phenyl rings at 2,6- positions of the piperidine ring occupy equatorial orientation as can be seen from the corresponding torsion angles [C4—C3—C2—C13=] 175.8 (1)° & [C4—C5—C6—C7=] -177.5 (1)°, respectively. The dihedral angle between the two phenyl rings is 64.22 (7)°. The ethyl group substituted at 3^rd^ position of the piperdine moiety is in equatorial oreintation.

The molecules are controlled by C—H ··· π type of intermolecular interactions in addition to van der Waals forces. The molecules are stacked one over the other while packing in the unit cell (Fig. 2).

### 2. Experimental

A mixture of piperidin-4-one (10 mM), and 80% hydrazine hydrate (3.1 ml) in diethylene glycol (100 ml) was heated on a steam bath for 2 h. Potassium hydroxide pellets (2.8 g) were added to the mixture and the contents were refluxed for another 2 h. The reaction mixture was cooled (Ponnuswamy *et al.*, 2002). The product formed was filtered and recrystallized from ethanol.

### 3. Refinement

C-bound H atoms were positioned geometrically (C–H = 0.93–0.98 Å) and allowed to ride on their parent atoms, with *U*_iso_(H) = 1.5*U*_eq_(C) for methyl H atoms and 1.2*U*_eq_(C) for all other H atoms. The N-bound H was located in a difference Fourier map and was refined with a distance restraint; its temperature factor was refined.

### Crystal data

**Table:**

C_19_H_23_N	*Z* = 2
*M**_r_* = 265.38	*F*(000) = 288
Triclinic, *P*1	*D*_x_ = 1.117 Mg m^−^^3^
Hall symbol: -P 1	Mo *K*α radiation, λ = 0.71073 Å
*a* = 5.5384 (5) Å	Cell parameters from 2579 reflections
*b* = 9.2717 (9) Å	θ = 1.3–26.5°
*c* = 16.0483 (14) Å	µ = 0.06 mm^−^^1^
α = 75.508 (5)°	*T* = 293 K
β = 89.474 (5)°	Block, colorless
γ = 81.625 (5)°	0.20 × 0.19 × 0.19 mm
*V* = 789.04 (13) Å^3^	

### Data collection

**Table:**

Bruker SMART APEXII CCD diffractometer	3251 independent reflections
Radiation source: fine-focus sealed tube	2579 reflections with *I* > 2σ(*I*)
Graphite monochromator	*R*_int_ = 0.025
ω and φ scans	θ_max_ = 26.5°, θ_min_ = 1.3°
Absorption correction: multi-scan (*SADABS*; Bruker, 2008)	*h* = −6→6
*T*_min_ = 0.987, *T*_max_ = 0.988	*k* = −11→8
11467 measured reflections	*l* = −20→20

### Refinement

**Table:**

Refinement on *F*^2^	Primary atom site location: structure-invariant direct methods
Least-squares matrix: full	Secondary atom site location: difference Fourier map
*R*[*F*^2^ > 2σ(*F*^2^)] = 0.043	Hydrogen site location: inferred from neighbouring sites
*wR*(*F*^2^) = 0.124	H atoms treated by a mixture of independent and constrained refinement
*S* = 1.03	*w* = 1/[σ^2^(*F*_o_^2^) + (0.0584*P*)^2^ + 0.1238*P*] where *P* = (*F*_o_^2^ + 2*F*_c_^2^)/3
3251 reflections	(Δ/σ)_max_ < 0.001
185 parameters	Δρ_max_ = 0.13 e Å^−^^3^
0 restraints	Δρ_min_ = −0.18 e Å^−^^3^

### Special details

**Table:**

Geometry. All e.s.d.'s (except the e.s.d. in the dihedral angle between two l.s. planes) are estimated using the full covariance matrix. The cell e.s.d.'s are taken into account individually in the estimation of e.s.d.'s in distances, angles and torsion angles; correlations between e.s.d.'s in cell parameters are only used when they are defined by crystal symmetry. An approximate (isotropic) treatment of cell e.s.d.'s is used for estimating e.s.d.'s involving l.s. planes.
Refinement. Refinement of *F*^2^ against ALL reflections. The weighted *R*-factor *wR* and goodness of fit *S* are based on *F*^2^, conventional *R*-factors *R* are based on *F*, with *F* set to zero for negative *F*^2^. The threshold expression of *F*^2^ > σ(*F*^2^) is used only for calculating *R*-factors(gt) *etc*. and is not relevant to the choice of reflections for refinement. *R*-factors based on *F*^2^ are statistically about twice as large as those based on *F*, and *R*- factors based on ALL data will be even larger.

### Fractional atomic coordinates and isotropic or equivalent isotropic displacement parameters (Å^2^)

**Table:**

	*x*	*y*	*z*	*U*_iso_*/*U*_eq_	
C2	0.1613 (2)	0.41471 (13)	0.23423 (7)	0.0434 (3)	
H2	0.0128	0.3751	0.2238	0.052*	
C3	0.3820 (2)	0.31957 (13)	0.20539 (8)	0.0471 (3)	
H3	0.5289	0.3604	0.2160	0.057*	
C4	0.4049 (3)	0.15761 (15)	0.26061 (9)	0.0594 (4)	
H4A	0.2685	0.1119	0.2470	0.071*	
H4B	0.5538	0.1004	0.2466	0.071*	
C5	0.4091 (3)	0.14914 (15)	0.35648 (9)	0.0582 (4)	
H5A	0.5578	0.1813	0.3719	0.070*	
H5B	0.4089	0.0456	0.3888	0.070*	
C6	0.1893 (2)	0.24831 (14)	0.38030 (8)	0.0484 (3)	
H6	0.0405	0.2116	0.3672	0.058*	
C7	0.1925 (2)	0.24838 (13)	0.47436 (8)	0.0471 (3)	
C8	0.0291 (3)	0.17969 (17)	0.53029 (9)	0.0612 (4)	
H8	−0.0888	0.1347	0.5095	0.073*	
C9	0.0372 (3)	0.17646 (19)	0.61675 (10)	0.0708 (4)	
H9	−0.0737	0.1285	0.6535	0.085*	
C10	0.2063 (3)	0.24306 (17)	0.64857 (9)	0.0639 (4)	
H10	0.2111	0.2413	0.7067	0.077*	
C11	0.3691 (3)	0.31261 (19)	0.59385 (10)	0.0721 (4)	
H11	0.4852	0.3585	0.6150	0.086*	
C12	0.3625 (3)	0.31531 (18)	0.50754 (9)	0.0663 (4)	
H12	0.4745	0.3630	0.4712	0.080*	
C13	0.1329 (2)	0.57950 (13)	0.18804 (7)	0.0429 (3)	
C14	0.3020 (2)	0.66778 (14)	0.20280 (8)	0.0501 (3)	
H14	0.4373	0.6233	0.2389	0.060*	
C15	0.2726 (3)	0.82000 (15)	0.16494 (9)	0.0596 (4)	
H15	0.3872	0.8774	0.1759	0.071*	
C16	0.0745 (3)	0.88777 (16)	0.11094 (9)	0.0657 (4)	
H16	0.0540	0.9909	0.0859	0.079*	
C17	−0.0922 (3)	0.80212 (17)	0.09430 (9)	0.0665 (4)	
H17	−0.2250	0.8472	0.0572	0.080*	
C18	−0.0642 (2)	0.64924 (16)	0.13235 (8)	0.0545 (3)	
H18	−0.1786	0.5924	0.1206	0.065*	
C19	0.3650 (3)	0.32871 (17)	0.10912 (9)	0.0609 (4)	
H19A	0.2269	0.2818	0.0983	0.073*	
H19B	0.3334	0.4339	0.0778	0.073*	
C20	0.5909 (3)	0.2544 (2)	0.07412 (11)	0.0823 (5)	
H20A	0.5665	0.2649	0.0136	0.124*	
H20B	0.6213	0.1494	0.1034	0.124*	
H20C	0.7282	0.3016	0.0831	0.124*	
N1	0.1873 (2)	0.40159 (11)	0.32687 (6)	0.0463 (3)	
H1	0.069 (3)	0.4663 (17)	0.3429 (10)	0.064 (4)*	

### Atomic displacement parameters (Å^2^)

**Table:**

	*U*^11^	*U*^22^	*U*^33^	*U*^12^	*U*^13^	*U*^23^
C2	0.0430 (6)	0.0478 (6)	0.0404 (6)	−0.0064 (5)	0.0015 (5)	−0.0133 (5)
C3	0.0495 (7)	0.0477 (7)	0.0462 (6)	−0.0048 (5)	0.0039 (5)	−0.0172 (5)
C4	0.0736 (9)	0.0475 (7)	0.0576 (8)	0.0000 (6)	0.0069 (7)	−0.0194 (6)
C5	0.0725 (9)	0.0444 (7)	0.0532 (8)	0.0003 (6)	0.0024 (6)	−0.0092 (6)
C6	0.0534 (7)	0.0488 (7)	0.0438 (6)	−0.0120 (5)	0.0022 (5)	−0.0106 (5)
C7	0.0521 (7)	0.0439 (6)	0.0435 (6)	−0.0052 (5)	0.0022 (5)	−0.0085 (5)
C8	0.0607 (8)	0.0709 (9)	0.0558 (8)	−0.0190 (7)	0.0106 (6)	−0.0180 (7)
C9	0.0776 (10)	0.0810 (10)	0.0539 (8)	−0.0192 (8)	0.0214 (7)	−0.0138 (7)
C10	0.0784 (10)	0.0673 (9)	0.0432 (7)	0.0027 (7)	0.0035 (7)	−0.0163 (6)
C11	0.0844 (11)	0.0823 (11)	0.0559 (8)	−0.0238 (9)	−0.0037 (8)	−0.0226 (8)
C12	0.0765 (10)	0.0774 (10)	0.0502 (8)	−0.0329 (8)	0.0065 (7)	−0.0137 (7)
C13	0.0443 (6)	0.0483 (6)	0.0359 (6)	−0.0011 (5)	0.0050 (5)	−0.0136 (5)
C14	0.0546 (7)	0.0511 (7)	0.0450 (6)	−0.0042 (5)	−0.0004 (5)	−0.0148 (5)
C15	0.0754 (9)	0.0524 (8)	0.0544 (8)	−0.0132 (7)	0.0103 (7)	−0.0180 (6)
C16	0.0847 (11)	0.0482 (7)	0.0547 (8)	0.0038 (7)	0.0147 (7)	−0.0037 (6)
C17	0.0634 (9)	0.0698 (9)	0.0517 (8)	0.0098 (7)	−0.0022 (6)	0.0007 (7)
C18	0.0489 (7)	0.0632 (8)	0.0477 (7)	−0.0033 (6)	−0.0004 (5)	−0.0101 (6)
C19	0.0698 (9)	0.0651 (8)	0.0499 (7)	−0.0034 (7)	0.0078 (6)	−0.0223 (6)
C20	0.0885 (12)	0.0953 (13)	0.0677 (10)	−0.0023 (10)	0.0219 (9)	−0.0361 (9)
N1	0.0537 (6)	0.0443 (6)	0.0391 (5)	−0.0011 (5)	0.0045 (4)	−0.0109 (4)

### Geometric parameters (Å, º)

**Table:**

C2—N1	1.4678 (14)	C10—H10	0.9300
C2—C13	1.5080 (16)	C11—C12	1.380 (2)
C2—C3	1.5383 (17)	C11—H11	0.9300
C2—H2	0.9800	C12—H12	0.9300
C3—C4	1.5287 (18)	C13—C14	1.3882 (17)
C3—C19	1.5289 (17)	C13—C18	1.3898 (17)
C3—H3	0.9800	C14—C15	1.3760 (18)
C4—C5	1.5209 (18)	C14—H14	0.9300
C4—H4A	0.9700	C15—C16	1.376 (2)
C4—H4B	0.9700	C15—H15	0.9300
C5—C6	1.5217 (19)	C16—C17	1.371 (2)
C5—H5A	0.9700	C16—H16	0.9300
C5—H5B	0.9700	C17—C18	1.382 (2)
C6—N1	1.4631 (15)	C17—H17	0.9300
C6—C7	1.5099 (16)	C18—H18	0.9300
C6—H6	0.9800	C19—C20	1.512 (2)
C7—C8	1.3773 (19)	C19—H19A	0.9700
C7—C12	1.3786 (19)	C19—H19B	0.9700
C8—C9	1.381 (2)	C20—H20A	0.9600
C8—H8	0.9300	C20—H20B	0.9600
C9—C10	1.362 (2)	C20—H20C	0.9600
C9—H9	0.9300	N1—H1	0.902 (16)
C10—C11	1.368 (2)		
			
N1—C2—C13	108.11 (9)	C11—C10—H10	120.4
N1—C2—C3	109.21 (10)	C10—C11—C12	120.54 (14)
C13—C2—C3	113.50 (10)	C10—C11—H11	119.7
N1—C2—H2	108.6	C12—C11—H11	119.7
C13—C2—H2	108.6	C7—C12—C11	121.02 (14)
C3—C2—H2	108.6	C7—C12—H12	119.5
C4—C3—C19	112.37 (10)	C11—C12—H12	119.5
C4—C3—C2	109.21 (10)	C14—C13—C18	117.86 (12)
C19—C3—C2	111.62 (10)	C14—C13—C2	120.10 (10)
C4—C3—H3	107.8	C18—C13—C2	122.01 (11)
C19—C3—H3	107.8	C15—C14—C13	121.05 (12)
C2—C3—H3	107.8	C15—C14—H14	119.5
C5—C4—C3	112.36 (10)	C13—C14—H14	119.5
C5—C4—H4A	109.1	C16—C15—C14	120.39 (14)
C3—C4—H4A	109.1	C16—C15—H15	119.8
C5—C4—H4B	109.1	C14—C15—H15	119.8
C3—C4—H4B	109.1	C17—C16—C15	119.47 (13)
H4A—C4—H4B	107.9	C17—C16—H16	120.3
C4—C5—C6	111.12 (11)	C15—C16—H16	120.3
C4—C5—H5A	109.4	C16—C17—C18	120.44 (13)
C6—C5—H5A	109.4	C16—C17—H17	119.8
C4—C5—H5B	109.4	C18—C17—H17	119.8
C6—C5—H5B	109.4	C17—C18—C13	120.78 (13)
H5A—C5—H5B	108.0	C17—C18—H18	119.6
N1—C6—C7	109.94 (10)	C13—C18—H18	119.6
N1—C6—C5	107.98 (10)	C20—C19—C3	114.38 (13)
C7—C6—C5	112.76 (10)	C20—C19—H19A	108.7
N1—C6—H6	108.7	C3—C19—H19A	108.7
C7—C6—H6	108.7	C20—C19—H19B	108.7
C5—C6—H6	108.7	C3—C19—H19B	108.7
C8—C7—C12	117.64 (12)	H19A—C19—H19B	107.6
C8—C7—C6	121.42 (11)	C19—C20—H20A	109.5
C12—C7—C6	120.93 (11)	C19—C20—H20B	109.5
C7—C8—C9	121.17 (14)	H20A—C20—H20B	109.5
C7—C8—H8	119.4	C19—C20—H20C	109.5
C9—C8—H8	119.4	H20A—C20—H20C	109.5
C10—C9—C8	120.50 (14)	H20B—C20—H20C	109.5
C10—C9—H9	119.8	C6—N1—C2	113.77 (9)
C8—C9—H9	119.8	C6—N1—H1	110.9 (10)
C9—C10—C11	119.13 (13)	C2—N1—H1	109.8 (10)
C9—C10—H10	120.4		
			
N1—C2—C3—C4	55.09 (13)	C10—C11—C12—C7	0.0 (3)
C13—C2—C3—C4	175.78 (10)	N1—C2—C13—C14	52.66 (14)
N1—C2—C3—C19	179.96 (10)	C3—C2—C13—C14	−68.65 (14)
C13—C2—C3—C19	−59.35 (14)	N1—C2—C13—C18	−125.03 (12)
C19—C3—C4—C5	−177.17 (12)	C3—C2—C13—C18	113.65 (13)
C2—C3—C4—C5	−52.74 (15)	C18—C13—C14—C15	1.34 (18)
C3—C4—C5—C6	53.94 (16)	C2—C13—C14—C15	−176.45 (11)
C4—C5—C6—N1	−55.80 (14)	C13—C14—C15—C16	−0.4 (2)
C4—C5—C6—C7	−177.47 (11)	C14—C15—C16—C17	−0.7 (2)
N1—C6—C7—C8	129.57 (13)	C15—C16—C17—C18	1.0 (2)
C5—C6—C7—C8	−109.88 (14)	C16—C17—C18—C13	0.0 (2)
N1—C6—C7—C12	−51.55 (16)	C14—C13—C18—C17	−1.11 (19)
C5—C6—C7—C12	69.00 (16)	C2—C13—C18—C17	176.64 (11)
C12—C7—C8—C9	−0.7 (2)	C4—C3—C19—C20	−64.41 (17)
C6—C7—C8—C9	178.18 (13)	C2—C3—C19—C20	172.50 (13)
C7—C8—C9—C10	0.7 (2)	C7—C6—N1—C2	−174.65 (10)
C8—C9—C10—C11	−0.3 (2)	C5—C6—N1—C2	61.95 (13)
C9—C10—C11—C12	−0.1 (2)	C13—C2—N1—C6	173.60 (10)
C8—C7—C12—C11	0.4 (2)	C3—C2—N1—C6	−62.46 (13)
C6—C7—C12—C11	−178.57 (14)		

### Hydrogen-bond geometry (Å, º)

Cg2 and Cg3 are the centroids of the C7–C12 and C13–C18 rings, respectively.

**Table:**

*D*—H···*A*	*D*—H	H···*A*	*D*···*A*	*D*—H···*A*
C5—H5*B*···*Cg*2^i^	0.97	3.14	3.893 (2)	136
C10—H10···*Cg*3^ii^	0.93	2.92	3.702 (2)	142
C20—H20*A*···*Cg*3^iii^	0.96	3.17	3.926 (2)	137

Symmetry codes: (i) −*x*+1, −*y*, −*z*+1; (ii) −*x*, −*y*+1, −*z*+1; (iii) −*x*+1, −*y*+1, −*z*.
